# Covid-19 pandemic and equity of global human papillomavirus vaccination: descriptive study of World Health Organization-Unicef vaccination coverage estimates

**DOI:** 10.1136/bmjmed-2023-000726

**Published:** 2024-01-29

**Authors:** Rebecca Mary Casey, Hiroki Akaba, Terri B Hyde, Paul Bloem

**Affiliations:** 1 Centers for Disease Control and Prevention, Atlanta, Georgia, USA; 2 Immunization, Vaccines and Biologicals Department, World Health Organization, Geneva, Switzerland

**Keywords:** COVID-19, Public health, Healthcare Disparities

## Abstract

**Objective:**

To analyse progress in global vaccination against human papillomavirus (HPV) during the covid-19 pandemic, with a particular focus on equity.

**Design:**

Descriptive study of World Health Organization-Unicef vaccination coverage estimates.

**Setting:**

WHO-Unicef estimates of global, regional, and national HPV vaccination coverage, before (2010-19) and during (2020-21) the covid-19 pandemic.

**Participants:**

Girls aged 9-14 years who received a HPV vaccine globally before (12.3 million in 2019) and during (2020-21) the covid-19 pandemic (10.6 million in 2021).

**Main outcome measures:**

Mean programme and population adjusted coverage for first dose HPV vaccine (HPV1) by country, country income (World Bank income categories), sex, and WHO region, before (2010-19) and during (2020-21) the covid-19 pandemic, based on WHO-Unicef estimates of HPV vaccination coverage. Annual number of national HPV vaccine programme introduced since the first HPV vaccine licence was granted in 2006, based on data reported to WHO-Unicef. Number of girls vaccinated before (2019) versus during (2020-21) the covid-19 pandemic period.

**Results:**

Mean coverage of HPV vaccination programmes among girls decreased from 65% in 2010-19 to 50% in 2020-21 in low and middle income countries compared with an increase in high income countries from 61% to 69% for the same periods. Population adjusted HPV1 coverage was higher among girls in high income countries before and during the covid-19 pandemic than in girls in low and middle income countries. During the covid-19 pandemic, population adjusted HPV1 coverage among boys in high income countries was higher and remained higher than coverage among girls in low and middle income countries. Globally, 23 countries recorded a severe reduction in their HPV programme (≥50% reduction in coverage), and another 3.8 million girls globally did not receive a HPV vaccine in countries with existing HPV vaccination programmes in 2020-21 compared with 2019. A reduction was seen in the annual rate of new introductions of national HPV vaccine programmes during 2020-21, affecting countries in all income categories, followed by an increase in introductions during 2022. During the second half of 2023, several low and middle income countries with large birth cohorts and a high relative burden of cervical cancer have yet to introduce HPV vaccination.

**Conclusions:**

Although HPV vaccines have been available for more than 15 years, global HPV vaccination coverage is low. During the covid-19 pandemic period (2020-21 globally), worsening coverage, delayed introductions of national vaccine programmes, and an increase in missed girls globally (ie, girls who did not receive a HPV vaccine compared with the previous year in countries with an existing HPV vaccination programme) that disproportionately affected girls in low and middle income countries were found. Urgent and innovative recovery efforts are needed to accelerate national introduction of HPV vaccination programmes and achieve high coverage of HPV vaccination worldwide.

**What is already known on this topic**

- A safe and effective vaccine against human papillomavirus (HPV) has been available since 2006, mainly targeted at girls aged 9-14 years, with the goal of reaching 90% vaccination coverage by 2030 as part of the global strategy to eliminate cervical cancer
- About two thirds of countries globally have introduced a HPV vaccine into their routine immunisation schedule

**What this study adds**

- Globally, HPV vaccination coverage was low and decreased during the covid-19 pandemic, and the rate of introduction of new HPV vaccination programmes was also reduced
- In 2021, only eight countries achieved HPV1 vaccination coverage ≥90% among target girls
- Inequities in coverage and introduction status of HPV vaccination by sex, country, country income category, and between and within World Health Organization regions were found
- HPV vaccination coverage among girls in low and middle income countries decreased from 65% before the covid-19 pandemic (2010-19) to 50% during the pandemic (2020-21)
- High income countries were better at maintaining HPV vaccination coverage during the covid-19 pandemic than low and middle income countries

**How this study might affect research, practice, or policy**

- Urgent and innovative recovery efforts are needed to accelerate national introductions and achieve high coverage for HPV vaccination globally
- Implementation research is needed to better understand best practices in delivery of HPV vaccination to achieve and sustain high coverage and identify approaches that can be tailored and implemented across different countries and settings

## Introduction

Cervical cancer is the fourth most common form of cancer among women worldwide, with >340 000 estimated deaths from cervical cancer annually.^1^ This cancer is caused by persistent infection with oncogenic genotypes of the human papillomavirus (HPV) and is preventable by a highly effective and safe vaccine that has been available since 2006. In August 2020, the World Health Assembly adopted the global strategy to eliminate cervical cancer as a public health problem by 2030.^2^ To eliminate cervical cancer, all countries must reach and maintain an incidence rate <4 per 100 000 women. Vaccination is one of three key strategies to achieve the goal of eliminating cervical cancer, with a target of vaccinating 90% of girls with the HPV vaccine by the age of 15 years. Most countries currently offer a one or two dose schedule for girls aged 9-14 years but variability exists by country in the primary target group and schedule. The other two strategies are screening and treatment of precancerous cervical lesions and cancer.

Progress has been made in expanding HPV vaccination globally since 2006, and about two thirds (127/194) of countries have introduced HPV vaccination (as of 2022). Gavi, the Vaccine Alliance (Gavi), a public-private sector alliance aiming to increase equitable and sustainable use of vaccines, has provided financial support to eligible countries (based on gross national income per capita) for the introduction of HPV vaccination since 2012. Many countries have yet to introduce HPV vaccination into their national immunisation programme, however, and in most countries where HPV vaccination is provided as part of the national immunisation schedule, coverage is lower than the target level of 90%.

Global coverage of HPV vaccination is lower than coverage for all other antigens in the World Health Organization recommended schedule.^3^ As well as the challenges of achieving and sustaining high coverage for vaccination, in many areas access to systematic screening and treatment for cervical cancer has not been widely implemented, and the burden of cervical cancer disproportionately affects low and middle income countries. About 90% of deaths from cervical cancer in 2021 occurred in low and middle income countries^1^; around 10% occurred in high income countries. The covid-19 pandemic has had a profound effect on communities and has disrupted the delivery of routine health services in many countries, including the provision of immunisation programmes. We analysed progress in global HPV vaccination during the covid-19 pandemic, with a particular focus on equity.

## Methods

We analysed mean coverage of HPV vaccination programmes by country, country income (based on World Bank income categories), sex, and WHO region for the periods before (2010-19) and during (2020-21) the covid-19 pandemic. Calculation of mean coverage was based on estimates of coverage of national HPV vaccination programmes (first dose (HPV1) and last dose of a complete HPV vaccination series) from the WHO-Unicef estimates of national coverage.^3^ Eight country specific coverage estimates (HPV vaccination programme coverage for the target population and HPV vaccination coverage by age 15 years, by sex, and first and last dose) are provided by WHO and Unicef every year after review of available coverage data, including the annual joint reporting form from countries. Data for sex are from WHO-Unicef estimates of national coverage rather than from patient reported gender. For analysis of data on mean coverage of HPV vaccination programmes, we excluded countries that have not yet introduced the vaccine, and therefore the number of countries included in the analysis varied by year.

We also calculated the population adjusted coverage for a single age cohort by sex, country, country income category, WHO region, and globally. Single age cohort is defined as a one year age cohort based on age at the last birthday. We used the national estimates for the population of adolescents from the United Nations Population Division, World Population Prospects 2022,^4^ as the target population for the single age cohort (denominator). Given that the target population for any single age cohort aged nine to 14 years is not expected to vary substantially, we used the population estimate for age 10 years for existing HPV vaccination programmes targeting a single age cohort, irrespective of the age specification of the country's HPV vaccine programme. We calculated the numerator as the total number of children in a single age cohort who were vaccinated in each country, based on the WHO-Unicef coverage estimate and target population for that country. For analysis of population adjusted coverage of HPV vaccination, we included all countries irrespective of the introduction status of the HPV vaccination programmes.

For our analysis, we categorised countries according to the relative change in coverage of HPV vaccination programmes between the end of the period before the covid-19 pandemic (2019) and the end of the pandemic (2021). We categorised limited change as <10% decrease in coverage, moderate reduction as 10-49% decrease in coverage, and severe reduction as ≥50% decrease in coverage. We analysed the number of girls who were vaccinated in each country for each single age cohort for each year between 2019 and 2021, as well as describing incremental changes. We obtained the introduction status of the HPV vaccine (including 2022) by country from the WHO HPV vaccine dashboard,^5^ to report the annual number of introductions of national vaccine programmes worldwide since the HPV vaccine was first introduced in 2006, including by country income category and by eligibility for financial support from Gavi. These data are mainly based on reports of introduction status by countries through the annual WHO-Unicef joint reporting form. The HPV vaccine dashboard, however, is also updated to reflect the most recent status of introduction of the HPV vaccine by country, based on information reported to WHO and partners.

Lastly, we compared the current introduction and coverage of HPV vaccine programmes with the global burden of cervical cancer, grouped by income category, to further examine the equity of HPV vaccination. Information about country income categories was obtained from the World Bank.^6^ Income categories were defined according to the criteria of the 2022 World Bank income categories for high income countries and low and middle income countries (low and middle income countries included low income countries, lower middle income countries, and upper middle income countries). We used data for disease burden of cervical cancer from the Global Cancer Observatory (GLOBOCAN) 2020.^7^ This online database is regularly updated by the International Agency for Research on Cancer. Categorisation of regions was based on WHO regional distribution.^8^ We categorised countries as Gavi eligible or non-Gavi eligible, depending on the criteria described in the Gavi eligibility and transition policy for the relevant year.^9^ Data analysis was conducted with R version 4.2.2.

### Patient and public involvement

Patients and/or the public were not involved in the design, or conduct, or reporting, or dissemination plans of this research, because we used routinely reported data for this analysis. Results have been disseminated through usual global immunisation partner channels.

## Results

**Figure 1:**
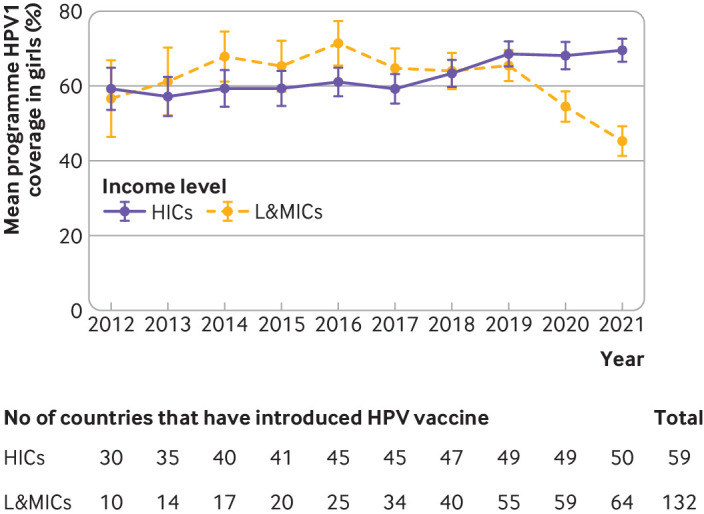
Mean programme coverage with first dose of human papillomavirus (HPV) vaccine (HPV1) among girls, by country income category, worldwide, 2012-21. Mean programme coverage calculation does not include countries without an existing HPV vaccination programme. Country income categories were based on criteria from 2022 World Bank income categories for high income countries (HICs) and low and middle income countries (L&MICs). Three countries did not have a World Bank income category and were excluded (Venezuela, Niue, and Cook Islands). Values are mean and 95% confidence interval

We found that mean coverage of HPV1 vaccination programmes has been trending upwards in high income countries overall since 2010 and the number of high income countries that have introduced HPV vaccination nationally has also been increasing (figure 1). In low and middle income countries, coverage of HPV1 vaccination programmes increased overall in 2010-19, peaking in 2016, when a number of countries reported very high coverage (≥95%), followed by a 6% reduction in 2016 that stabilised in 2017-19. Mean coverage of HPV1 vaccination programmes among target girls decreased from 65% during the period before the covid-19 pandemic (2010-19) to 50% during the pandemic (2020-21) in low and middle income countries, compared with an increase in high income countries from 61% to 69% for the same periods. Before the pandemic, mean programme coverage for girls was higher in low and middle income countries compared with high income countries.

**Figure 2:**
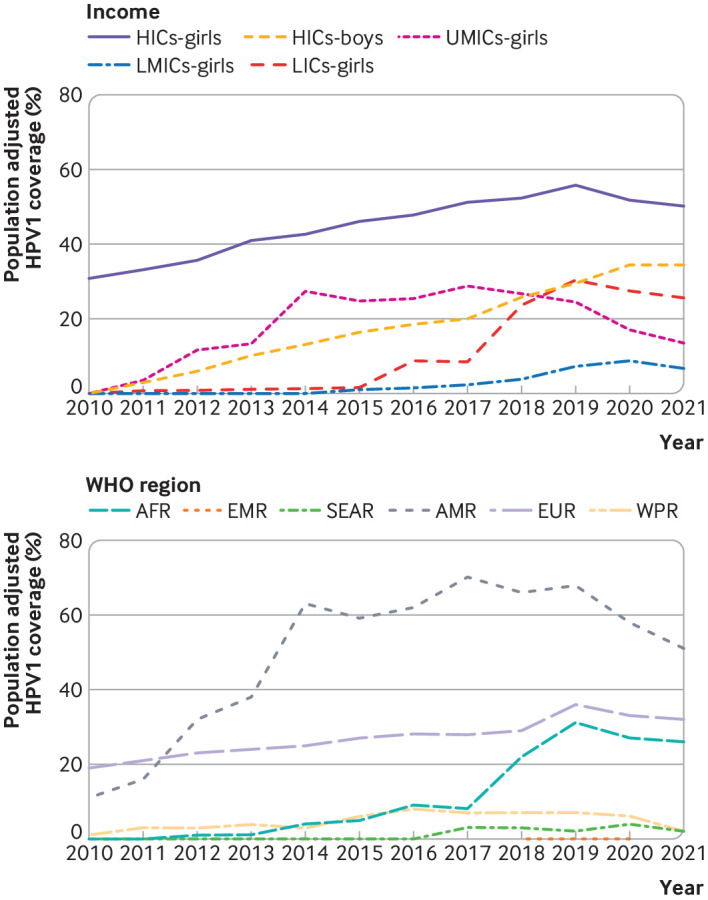
(Top) Population adjusted coverage for first dose of human papillomavirus (HPV) vaccine (HPV1), by country income category and sex, worldwide, 2010-21. HICs-girls=coverage in girls from high income countries; HICs-boys=coverage in boys from high income countries; UMICs-girls=coverage in girls from upper middle income countries; LMICs-girls=coverage in girls from lower middle income countries; LICs-girls=coverage in girls from low income countries. Country income categories were based on criteria of 2022 World Bank income categories for low income, lower middle income, upper middle income, and high income countries. Three countries did not have a World Bank income category and were excluded (Venezuela, Niue, and Cook Islands). (Bottom) Population adjusted coverage with HPV1 among girls by World Health Organization region, worldwide, 2010-21. AFR=African region; AMR=Americas region; EMR=Eastern Mediterranean region; EUR=European region; SEAR=South East Asia region; WPR=Western Pacific region

Population adjusted HPV1 coverage among girls was highest among those in high income countries in 2010-21 compared with girls from other country income categories (figure 2). Coverage among boys in high income countries has been gradually increasing since 2010. During the covid-19 pandemic, population adjusted HPV1 coverage among boys in high income countries was higher and remained higher than that in girls from low and middle income countries (ie, upper middle income, lower middle income, and low income countries).

Overall, the number of gender neutral HPV vaccination programme introduced increased from 0 to 48 between 2010 and 2022. As of July 2023, no low income country has a gender neutral vaccination programme, compared with 37/58 (64%), 9/52 (17%), and 1/53 (2%) in high income, upper middle income, and lower middle income countries, respectively. Also, a gender neutral HPV vaccine programme was introduced in 2019 in a country with no income categorisation by the World Bank (Niue). During the covid-19 pandemic, population adjusted HPV1 coverage among girls in upper middle income countries was proportionally more affected, with a decrease from 24% before the pandemic (2019) to 14% at the end of the pandemic (2021) for this analysis compared with the decrease in coverage among girls in high income countries (6%) and low income countries (4%).

We found no net change in coverage among girls in lower middle income countries between 2019 and 2021; however, coverage in lower middle income countries was the lowest across all country income categories before and during the covid-19 pandemic (2010-21). In 2021, HPV1 (first dose) vaccination coverage of ≥90% was achieved in eight (4%) countries, but only four (2%) countries globally achieved ≥90% with their complete HPV vaccination series. Among regions, population adjusted coverage was highest globally in the Americas region since 2012, but regional coverage also decreased the most from 68% in 2019 to 51% in 2021, which was mainly explained by reduced coverage in Mexico from 94% to 1% (figure 2). Overall, population adjusted HPV1 coverage has increased in the African region since the first introduction in 2011 (Rwanda), and a marked improvement in coverage was found from 8% in 2017 to 31% in 2019, followed by a reduction to 26% in 2021.

By region, HPV vaccination has yet to be introduced in 4/35 (11%), 9/53 (17%), 6/27 (22%), 21/47 (45%), 5/11 (45%), and 17/21 (81%) countries in the Americas, European, Western Pacific, African, South East Asian, and Eastern Mediterranean regions, respectively (as of July 2023). During the covid-19 pandemic, 23 countries globally reported a severe reduction in coverage of national HPV vaccination programmes (≥50% reduction in coverage). Two countries in the WHO European region recorded a severe reduction compared with 11 countries in the Americas region and seven countries in the African region. Globally, 23 countries did not report HPV vaccination coverage in 2021 compared with only nine countries in 2019.

**Figure 3:**
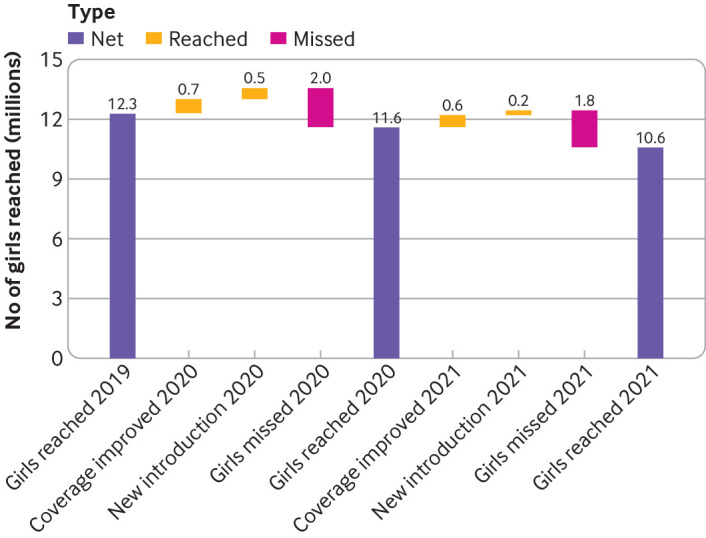
Global incremental gains and losses in number of girls who were vaccinated with first dose of human papillomavirus (HPV) vaccine (HPV1) by year, 2019-21. Girls reached=number of girls who were reported to have received at least one dose of HPV vaccine; coverage improved=number of additional girls who were vaccinated with at least one dose of HPV vaccine in countries with existing HPV programmes as a result of improvement in coverage; new introduction=number of additional girls reached with at least one dose of HPV vaccine in countries that introduced a new HPV vaccine programme during that calendar year; girls missed=number of girls who did not receive a HPV vaccine compared with the previous year in countries with an existing HPV vaccination programme

During the covid-19 pandemic, the number of girls worldwide who received a HPV vaccine increased both by improved coverage in some countries (1.3 million more girls) and by the introduction of national vaccine programmes (0.7 million more girls received a vaccine) in 2021 compared with 2019 (figure 3). Reduced HPV1 coverage in existing HPV programmes during the covid-19 pandemic (2020-21), however, resulted in 3.8 million more girls being missed globally. This finding gave an overall reduction in the total number of girls who received a HPV vaccine globally from 12.3 million in 2019 to 10.6 million in 2021.

**Figure 4:**
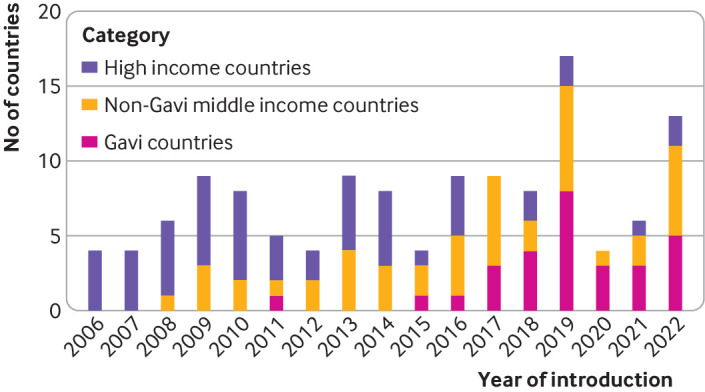
Number of countries that introduced national human papillomavirus (HPV) vaccine programmes, grouped by income category (based on 2022 World Bank income categories) and eligibility for financial support from Gavi, the Vaccine Alliance (Gavi), worldwide, 2006-22. Non-Gavi middle income countries=middle income countries that were not eligible for Gavi (following criteria for the relevant year); Gavi countries=countries that met Gavi eligibility criteria in the year of introduction. Eligibility threshold was adjusted for inflation on an annual basis

**Figure 5:**
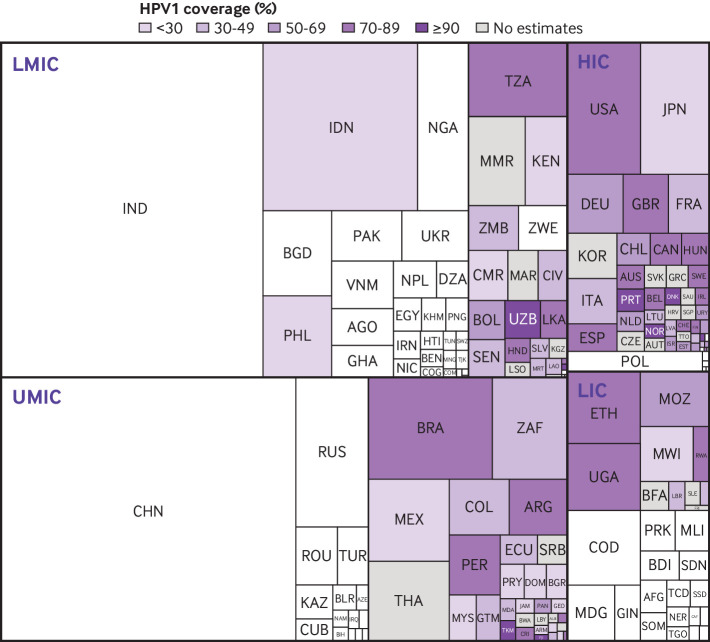
Burden of cervical cancer relative to coverage estimates of first dose of human papillomavirus (HPV) vaccine (HPV1), by country and income category, worldwide, 2021: high income (HIC), upper middle (UMIC), lower middle (LMIC), and low (LIC) income country. Size of squares is proportional to annual number of new patients with cervical cancer.^7^ Country income categories were based on criteria of 2022 World Bank income categories for low income, lower middle income, upper middle income, and high income countries. Three countries did not have a World Bank income category and were excluded (Venezuela, Niue, and Cook Islands). HIC: AUS=Australia; AUT=Austria; BEL=Belgium; CAN=Canada; CHL=Chile; CZE=Czech Republic; EST=Estonia; FIN=Finland; FRA=France; DEU=Germany; GRC=Greece; HRV=Croatia; HUN=Hungary; IRL=Ireland; ISR=Israel; ITA=Italy; JPN=Japan; LTU=Lithuania; LVA=Latvia; NLD=Netherlands; NOR=Norway; POL=Poland; PRT=Portugal; KOR=Republic of Korea; SAU=Saudi Arabia; SGP=Singapore; SVK=Slovakia; ESP=Spain; SWE=Sweden; TTO= Trinidad and Tobago; GBR=UK. URY=Uruguay. UMIC: ALB= Albania; ARG=Argentina; ARM=Armenia; AZE=Azerbaijan; BLR=Belarus; BIH=Bosnia and Herzegovina; BWA=Botswana; BRA=Brazil; BGR=Bulgaria; CHN=China; COL=Colombia; CRI=Costa Rica; CUB=Cuba; DOM=Dominican Republic; ECU=Ecuador; FJI= Fiji; GEO= Georgia; GTM=Guatemala; IRQ=Iraq; JAM=Jamaica; KAZ=Kazakhstan; LBY= Libya; MDA=Republic of Moldova; MYS=Malaysia; MEX=Mexico; NAM=Namibia; PAN= Panama; PRY=Paraguay; PER=Peru; ROU=Romania; RUS=Russia; SRB=Serbia; ZAF=South Africa; THA=Thailand; TKM= Turkmenistan; TUR=Turkey. LMIC: DZA=Algeria; AGO=Angola; BGD=Bangladesh; BEN=Benin; BOL=Bolivia (Plurinational State of); KHM=Cambodia; CMR=Cameroon; COG=Republic of Congo-Brazzaville; COM= Comoros; CIV=Côte d'Ivoire; EGY=Egypt; SLV=El Salvador; GHA=Ghana; HTI=Haiti; HND=Honduras; IND=India; IDN=Indonesia; IRN=Iran (Islamic Republic of); KEN=Kenya; KGZ=Kyrgyzstan; LAO= Lao People's Democratic Republic; LSO=Lesotho; MAR=Morocco; MMR=Myanmar; MNG=Mongolia; MRT=Mauritania; NPL=Nepal; NIC=Nicaragua; NGA=Nigeria; PAK=Pakistan; PNG=Papua New Guinea; PHL=Philippines; SEN=Senegal; LKA=Sri Lanka; SWZ= Eswatini; TJK=Tajikistan; TUN=Tunisia; TZA=United Republic of Tanzania; UKR=Ukraine; UZB=Uzbekistan; VNM=Vietnam; ZMB=Zambia; ZWE=Zimbabwe. LIC: AFG=Afghanistan; BFA=Burkina Faso; BDI=Burundi; TCD=Chad; PRK=Democratic People’s Republic of Korea; CAF=Central African Republic; COD=Democratic Republic of the Congo; ERI= Eritrea; ETH=Ethiopia; GIN=Guinea; LBR=Liberia; MDG=Madagascar; MWI=Malawi; MLI=Mali; MOZ=Mozambique; NER=Niger; RWA=Rwanda; SDN=Sudan; SLE= Sierra Leone; SOM=Somalia; SSD=South Sudan; TGO=Togo; UGA=Uganda

Most high income countries introduced the HPV vaccine nationally in the decade following the first availability of the HPV vaccine in 2006 (figure 4). From 2006 to 2016, 45 high income countries introduced the vaccine compared with 24 non-Gavi eligible middle income countries and only two Gavi eligible low or middle income countries. During the covid-19 pandemic, we found a large reduction in the number of countries, regardless of income category, introducing HPV vaccination. Overall, 18 HPV vaccine programmes were introduced in 2019, four in 2020, six in 2021, and 13 in 2022. The rate of Gavi eligible countries introducing national HPV vaccination by year increased from four countries in 2018 to eight in 2019, before decreasing to three in 2020. Three and five HPV vaccine programmes were introduced in Gavi eligible countries in 2021 and 2022, respectively. Of the 10 new introductions of national HPV vaccine programmes during the pandemic period, eight countries reported the introduction year coverage (mean 59%). Before the pandemic (2010-19), 63 of 83 countries that introduced HPV vaccination reported introduction year coverage (mean 62%). Several low and middle income countries with high annual numbers of patients with cervical cancer have yet to introduce HPV vaccination or vaccinate in select subnational geographies, including China, India, Russia, and the Democratic Republic of the Congo (figure 5). Among high income countries, several countries with a high disease burden have HPV1 coverage <70%, including Japan, France, and Italy.

## Discussion

### Principal findings

Globally, coverage of HPV vaccination programmes is low, and coverage decreased during the covid-19 pandemic. In 2021, only eight countries achieved HPV1 vaccination coverage among target girls of ≥90%, the global goal to eliminate cervical cancer. We found inequities in all aspects analysed, including differences in coverage and introduction status by country, country income category, and between and within WHO regions. High income countries were better at maintaining programme coverage during the covid-19 pandemic than low and middle income countries. This finding is likely in part a reflection of resources for health systems, capacity of immunisation programmes to manage the additional burden of covid-19 vaccination and outbreak response, communication strategy, organised outreach and catch-up activities for HPV vaccination, and trust and resilience of immunisation programmes during a period of adversity.

In common with other routine antigens, HPV vaccination has been disrupted by the competing priorities of controlling the covid-19 pandemic and the covid-19 vaccination response in diverse contexts globally. Similar findings on the effect of the covid-19 pandemic on HPV vaccination in different countries have been reported.^10–14^ In 68 of 116 countries worldwide that had introduced the HPV vaccine by the end of 2021, vaccination was delivered through schools^5^ which helps explain the severity of the reduction in coverage of HPV vaccination programmes globally because schools were closed during this period.

Despite a rapid fall in introductions of HPV vaccination at the start of the covid-19 pandemic, 10 countries introduced the HPV vaccine nationally during the covid-19 pandemic period, but only two managed to achieve HPV vaccination coverage ≥90%, and none sustained high coverage for >1 year. Encouragingly, the number of national introductions of HPV vaccination globally in countries from any income category recovered to 13 introductions in 2022, which could be a sign of recovery of immunisation programmes. Moreover, much of the success of the global HPV programme in reaching global coverage targets will depend on the HPV vaccine being successfully introduced in the most populous countries in the world where introduction of HPV vaccination is still pending and where large birth cohorts of girls are currently unprotected from cervical cancer in the highest prevalence settings. This success will also depend on a sustained increased global supply of vaccine, which has also been a challenge to global HPV vaccination for several years.

Overall, the number of gender neutral vaccination programmes by 2022 represents remarkable progress in equity by sex in providing the benefits of HPV vaccination, but this finding also highlights another inequity, likely related to domestic resources and the cost of the vaccine. Gavi funding has never been available to fund gender neutral programmes, and hence high income and upper middle income countries were generally the first to introduce HPV vaccines because of the greater availability of domestic funding. Also, because most (90%) HPV related cancers are among women (most of which are cervical cancers),^15^ global partners have supported focusing first on reaching the primary target of girls aged 9-14 years globally, a target that has not yet been reached. WHO recommended vaccination of secondary target populations (eg, girls aged ≥15 years and boys) only if feasible and affordable, and if resources are not diverted from vaccination of the primary target population or effective cervical cancer screening programmes.^16^ Vaccination of boys, however, has been recommended in many high income countries.

### Policy implications

Urgent recovery activities must be planned and implemented to ensure that missed girls (ie, those who did not receive a HPV vaccine in countries with an existing HPV vaccination programme) are vaccinated. As part of the ambitious goal of the Gavi HPV programme revitalisation initiative to increase the number of girls who receive a HPV vaccine from 9.8 million (2021) to >80 million by 2025, targeted assistance will be available to eligible countries to accelerate introductions and improve coverage. Additional support will be available for expanded multi-age cohort vaccination, targeting all girls and women aged 9-18 years in countries that have not implemented multi-age cohort vaccination previously.^17^ The Gavi approach for middle income countries^18^ will also provide critically needed support for countries that have fallen behind in HPV introductions compared with high income countries. This gap seems to have widened during the covid-19 pandemic.

Implementation research is needed to better understand best practices in delivery of HPV vaccination to achieve and sustain high coverage and identify approaches that can be tailored and implemented across different countries. Leveraging other opportunities for HPV vaccination to improve coverage (eg, co-delivery with other adolescent health interventions) and establishing adolescent health platforms will be important in achieving sustainable integrated delivery. The improving forecast for the supply of HPV vaccine^19^ and the alternative recommendation for a one dose schedule^16^ are timely opportunities, which together can stimulate recovery of programmes by allowing multi-age cohort catch-up campaigns, and identifying new efficiencies in delivery strategies for the HPV vaccine. Experience with other vaccines has shown that vaccination programmes with fewer doses are associated with improved rates for completion of the course and a lower logistical and economic burden.^20^

### Limitations of this study

Limitations of this study include the lack of details on context for individual countries to further explore the association and causality between the covid-19 pandemic and change in programme coverage during the pandemic period. Also, we did not have access to subnational data that would have provided a deeper understanding of the effect across countries, including potential equity challenges between different geographies, rural or urban settings, and population groups. Other studies similarly found widening inequalities in adolescent HPV vaccination at the subnational level during the covid-19 pandemic.^21^ Finally, estimates of WHO-Unicef coverage of HPV vaccination are mostly based on administrative data reported by countries that have variable accuracy, depending on the circumstances of that country. Variable reporting of national coverage data by countries throughout the reviewed period might have introduced bias. In particular, disruptions in HPV coverage caused by covid-19 in high income countries could be under-reported because proportionally more high income countries rely on frequent surveys that introduce a certain time delay. Others reported that their estimates of coverage based on surveys might over-represent HPV vaccination coverage because of the reporting behaviour of some groups of surveyed parents. Ongoing analyses of coverage data will be useful to understand and monitor recovery of HPV programmes.

### Conclusions

Although HPV vaccines have been available for >15 years, global coverage of HPV vaccination remains low. The covid-19 pandemic affected health and routine immunisation systems. During the covid-19 pandemic period globally, we found worsening coverage of HPV vaccination, delayed introductions of national vaccine programmes, and an increase in girls missed for vaccination (ie, the number of girls who did not receive a HPV vaccine compared with the previous year in countries with an existing HPV vaccination programme) that disproportionately affected girls in low and middle income countries. Urgent recovery efforts are needed to accelerate national introductions and achieve high coverage of HPV vaccination globally. Targeted investment and programme activities will be needed to ensure HPV vaccination reaches girls, including those at the highest risk for cervical cancer. Implementation research is needed to better understand successful sustainable delivery strategies.

## Data Availability

Data are available upon reasonable request.
